# Combining Cryptography with EEG Biometrics

**DOI:** 10.1155/2018/1867548

**Published:** 2018-05-22

**Authors:** Robertas Damaševičius, Rytis Maskeliūnas, Egidijus Kazanavičius, Marcin Woźniak

**Affiliations:** ^1^Department of Software Engineering, Kaunas University of Technology, Studentų 50-415, Kaunas, Lithuania; ^2^Centre of Real Time Computer Systems, Kaunas University of Technology, K. Baršausko 59-A338, Kaunas, Lithuania; ^3^Institute of Mathematics, Silesian University of Technology, Kaszubska 23, 44-100 Gliwice, Poland

## Abstract

Cryptographic frameworks depend on key sharing for ensuring security of data. While the keys in cryptographic frameworks must be correctly reproducible and not unequivocally connected to the identity of a user, in biometric frameworks this is different. Joining cryptography techniques with biometrics can solve these issues. We present a biometric authentication method based on the discrete logarithm problem and Bose-Chaudhuri-Hocquenghem (BCH) codes, perform its security analysis, and demonstrate its security characteristics. We evaluate a biometric cryptosystem using our own dataset of electroencephalography (EEG) data collected from 42 subjects. The experimental results show that the described biometric user authentication system is effective, achieving an Equal Error Rate (ERR) of 0.024.

## 1. Introduction

Brain computer interface (BCI) is a highly growing field of research with application in healthcare systems (from fall prevention to neuronal rehabilitation) to educational, self-regulation, production, marketing, and security as well as games and entertainment. BCI aims to provide a channel of communication that does not depend on the usual use of peripheral nerves and muscles [1]. While the main intended target application for BCI research is the development of motor function independent prosthetic devices for impaired patients, other applications of BCI, such as those for learning [2], gaming [3, 4], or entertainment [5], raise the need for ensuring the security and privacy of subjects using BCI systems. BCI systems are based on measurement of brain activity on the surface (in case of noninvasive BCI) or inside (in case of invasive BCI) of the human skull using electrodes. The results of the measurement represent the sum of electrical impulses emitted by a large number of brain's neurons. Noninvasive EEG signal is recorded by attaching the electrodes to the head of a subject according to a given map such as the 10–20 international system for the placement of EEG electrodes.

Recently, BCI applications for biometrics have attracted increased attention from the researchers. Biometrics provides means for identifying people based on their physiological characteristics [6]. Recently, there has been tremendous growth in research on cryptography and biometric frameworks because of incredible need for data security in numerous applications, such as e-commerce, e-health, e-government, e-voting, blockchain, law enforcement, digital forensics, and homeland security. The goal is to verify the identity of a subject using some characteristic of a subject. In cryptographic frameworks, users use their passwords or secret keys to protect their confidential data. However, the use of passwords for identification has some well-known drawbacks: textual passwords can be spied over or cracked, and secret keys are too long and difficult to memorize and can be stolen if stored somewhere. The downside of cryptography is that verification strategies are not unequivocally connected to the person identity. Unlike cryptography based authentication methods, biometrics using behavioural and physiological characteristics such as iris, fingerprints, electroencephalography (EEG) data, face, palm, voice, and gait, is convenient and cannot be forgotten or lost.

The EEG-based subject identification is relatively new. The advantages of using EEG for biometrics are its low exposability (cannot be casually obtained or stolen by external observers) and resistance to forced extraction because under-stress brain activity changes [7]. They also can be used by disabled patients or users missing some physical trait. Efforts to develop biometric methods and systems based on the EEG have targeted the development of subject condition monitoring tools, for example, for detection of sleep apnea [8], schizophrenia [9], or epilepsy [10]; the creation of BCI systems to assist disabled people [11]; and marketing [12]. Analysts predict that the global EEG and electrocardiography (ECG) biometrics market is to expand at a compound annual growth rate of 12.37% during the period 2016–2020 [13].

The suitability of using EEG for privacy and security applications can be attributed to morphological, anatomical, and functional plasticity (behaviour-related lasting changes in functional connections) traits [14], which contribute to discriminability between subjects [15]. Several studies (mainly from the fields of human physiology and genetics) have confirmed that the spectral characteristics of the EEG alpha waves (in the 8–12 Hz range, which reflect relaxation and disengagement) and the beta waves (in the 12–30 Hz range, related to action and concentration) of EEG show the strongest heritability relationship [16].

The difficulties related to using EEG data are its instability over time (the EEG permanence problem [17]). It is still difficult to achieve high accuracy of EEG-based biometric systems, which motivate researchers to explore new EEG data analysis methods. However, the research community still lacks knowledge on specific discriminant features of EEG suable for biometry [18]. Up to now, the EEG power spectrum features were used to achieve relatively good classification performance [18]. Several methods, which focus on the concepts and methods adopted from the network science, such as functional connectivity [19] and network organization [20], have been proposed. Fuzzy commitment (FC) scheme [21] can be used as a theoretical background for combining cryptography and biometrics. In the FC scheme, a secret key is linked to the reference biometric template, and the difference vector is calculated in such way that the secret key may be restored using the difference vector and the query biometric template. Another approach is a fuzzy vault (FV) based on polynomial reconstruction [22]. The FC and FV schemes have been applied to biometrics before [23, 24].

Here we propose a secure EEG-based cryptographic authentication scheme based on the commitment scheme adopted from [25], provide a theoretical analysis of the security characteristics of the proposed scheme, apply the scheme to biometric systems to construct a biometric cryptosystem using EEG signals, and evaluate it using our own dataset recorded from 42 subjects. The rest of the paper is organized as follows. In Section 2 we present the state-of-the-art overview of related work in EEG biometrics. We describe the proposed method in Section 3. We state theorems regarding the security characteristics of the cryptographic system in Section 4. We describe the application of the method on EEG dataset in Section 5. We present the experimental results and their evaluation in Section 6. Finally, the conclusions are given in Section 7.

## 2. State of the Art

Cognitive biometrics [26] uses brain signals as the source of information for user identification (authentication). User authentication is a process that ensures and confirms a user's identity in security systems. Using EEG signals for user authentication can be effective with varying degrees of accuracy. For example, Fladby [27] used power spectral features of alpha, beta low, beta high, and theta bands from just one EEG channel of 12 subjects performing eight different tasks (from simple relaxation to counting and reading) and a custom feature based distance metric for subject discrimination, achieving an EER of 21.42%. Palaniappan [28] used gamma band of visually evoked potential (VEP) signals and the neural network (NN) classifier to identify 20 individuals with an average accuracy of 99.06%.

Liang et al. [29] extracted the AR features from 8 EEG channels and used Support Vector Machine (SVM) to achieve an accuracy of 45.52% to 54.96% for subject separation task and an accuracy of 48.41% to 56.07% for subject identification task. Marcel and Millán [30] implemented a Gaussian mixture model (GMM) with maximum a posteriori (MAP) estimation for 9 subjects, achieving a half total error rate (HTER) of 6.6%.

Hema et al. [31] adopted feed forward NN for EEG using Power Spectral Density (PSD) features from EEG beta waves and reached an average accuracy of 94.4 to 97.5% on 6 subjects. He et al. [32] used a naïve Bayes (NB) classifier with autoregressive (AR) features and achieved a HTER of 6.7% for 4 subjects.

Mu and Hu [33] used the back-propagation NN on data derived from 6 channels of 3 subjects and achieved an 80.7% to 86.7% accuracy. Brigham and Kumar [34] used linear SVM classifier with the AR features and achieved accuracy of 98.96% on 122 subjects tested. Hu [35] used the NN on seven EEG signal features and obtained an 80% to 100% true acceptance rate (TAR) and a 0 to 30% false acceptance rate (FAR), while using data received from only 3 subjects.

Zúquete et al. [36] demonstrated the stability of EEG biometrics using visual stimulus to measure visual evoked potentials (VEP) and a combination of one-class classifiers (OCCs), including *k*-Nearest Neighbor (kNN) and Support Vector Data Description (SVDD). Ashby et al. [37] used linear SVM with AR and spectral characteristics of EEG signals from 14 EEG channels and achieved 2.4% to 5.1% false rejection rate (FRR) and 0.7% to 1.1% FAR for 5-subject authentication. Shedeed [38] used the NN on features obtained by fast Fourier transform (FFT) and wavelet packet decomposition (WPD) from 4 channels, achieving a 66% to 93% correct classification rate (CCR) using data from 3 subjects.

Chuang et al. [39] recorded single-channel EEG signals when a subject performs a custom task (e.g., singing or moving finger). The authentication system analyses the similarity between such brain data and training data to authenticate subjects, reaching about 99% accuracy. Yeom et al. [40] used Gaussian kernel SVM on the signal difference and time derivative features from 18 EEG channels and managed to achieve the accuracy around 86% on 10 subjects.

Dan et al. [41] used the polynomial kernel SVM based on the AR model parameters calculated on the EEG signal, recorded a single EEG channel, and obtained an accuracy of 65% to 75% on 13 subjects.

Delpozo-Banos et al. [18] used the functional connectivity patterns to represent effective features for improving EEG-based biometric systems and classification using Convolutional Neural Network (CNN) and achieved 97.5% accuracy in eyes-closed (EC) and 96.26% in eyes-open (EO) resting state conditions states when fusing PSD information from the parietooccipital (centroparietal in EO) parts of the brain of 10 subjects.

Abo-Zahhad et al. [42] achieved more than 99% authentication accuracy by using single-channel EEG signals from 10 and 15 subjects. Koike-Akino et al. [43] achieved 72% accuracy for 25-subject identification from EEG using a single 800 ms epoch and partial least-squares (PLS) dimensionality reduction method applied before quadratic discriminant analysis (QDA) classification.

Crobe et al. [44] obtained good results in the EEG gamma (EER = 0.131 and AUC = 0.943 in EO condition; EER = 0.130 and AUC = 0.933 in EC condition) and high beta (EER = 0.172 and AUC = 0.905 in EO condition; EER = 0.173 and AUC = 0.906 in EC condition) frequency bands.

Several studies presented the fusion of EEG with other modalities to get a multimodal biometric system such as in [45, 46]. Also see a survey of security and privacy challenges in BCI applications in [47]. EEG-based authentication was also considered as a part of smart driving systems to verify the driver's identity on demand [48]. However, using EEG brainwaves for authentication might result in risks for the privacy of users. For example, authors in [49] propose an authentication system that verifies an individual EEG signal when a subject performs a custom task. They also design an attack model by impersonating the thoughts of subjects to test the robustness of the authentication system. An adversary also can attack the authentication system via synthetic EEG signals, which are generated using a model based on the historical EEG data from a subject [50].

## 3. Description of EEG Biometry Method

First, we provide definitions required for understating of the biometric authentication method as given in [25].

**Definition 1 (discrete logarithm).**

Let *G* be a finite cyclic group of order *n*. Let *g* be a generator of *G* and let *h* ∈ *G*. The discrete logarithm of *h* to the base *g*, log_*g*_*h*, is the unique integer *u*, 0 ≤ *u* ≤ *n* − 1, such that *h* = *g*^*u*^.

**Definition 2 (discrete logarithm problem (DLP)).**

Given a prime number *p*, a generator of *g* of *z*_*p*_^*∗*^, and an element *h* ∈ *z*_*p*_^*∗*^, find the integer *u* such that *h* = *g*^*u*^  (mod *p*).

**Definition 3 (block code).**

A block code *C*(*n*, *k*) over an alphabet *A*^*∗*^ of *w* symbols is a set of *w*^*k*^  *n*-vectors called codewords. Associated with the code is an encoder {0,1}^*k*^ → *C* which maps a message *M*, a *k*-tuple, to its associated codeword.

**Definition 4 (decoding function).**

Let *C*(*n*, *k*) be a block code set with *w* = {0,1}. A decoding function *f*_*d*_ : {0,1}^*n*^ → *C* ∪ *ε* maps a message *c*′, a *n*-tuple, to correct codeword *c*, if *c*′ and *c* are sufficiently close according to appropriate metric. Otherwise, it maps it to invalid codeword *ε*.

**Definition 5 (hamming distance).**

Given code set *C*(*n*, *k*), the Hamming distance between two words *c*_*i*_ and *c*_*j*_ from the code set *C* is given by

(1) $$\begin{array}{c} H\left(\;c_{i},c_{j}\;\right)=\frac{1}{n}\overset{n}{\underset{r=1}{\sum }}\left|\;c_{i}^{r}-c_{j}^{r}\;\right|. \end{array}$$

**Definition 6 (error correction threshold).**

Error correction threshold *t*_*σ*_ of the error-correcting code *C*(*n*, *k*) is the largest number of errors that can be corrected in the corrupted codeword.

**Definition 7 (statistical distance).**

Let *X*_1_ and *X*_2_ be two random variables over the same space Ψ, and let *P*_1_ and *P*_2_ be their discrete probability distribution functions (PDFs). Then, the statistical distance between *P*_1_ and *P*_2_ is as follows:

(2) $$\begin{array}{c} D\left(\;P_{1},P_{2}\;\right)=\underset{\psi \in \Psi }{\sum }\left|\;Pr\left(\;X_{1}=\psi \;\right)-Pr\left(\;X_{2}=\psi \;\right)\;\right|. \end{array}$$

**Definition 8 (Bose-Chaudhuri-Hocquenghem (BCH) codes).**

Let *α* be a primitive element of Galois field GF(*q*^*m*^). For any positive integer *i*, let *m*_*i*_(*x*) be the minimal polynomial of *α*_*i*_ over GF(*q*^*m*^). The generator polynomial of the BCH code is defined as the least common multiple *P*(*x*) = lcm(*m*_1_(*x*),…, *m*_*d*−1_(*x*)).

The method, proposed by [25] and adopted here for EEG biometry, consists of three procedures: (1)* Setup*, which outputs a public key, (2)* Commit*, which takes as input and the message and outputs commitment to be sent and the opening value to be used for message verification, and (3)* Open*, which outputs true if verification succeeds or false otherwise. Three actors participate: the sender* Alice*, the receiver* Bob*, and the trusted third party* Trent*, who generated system parameters and publishes it to Alice and Bob parties.

Let *M* be the space of messages to commit to. The first stage is Setup stage (see Algorithm 1), where Trent generates and sends the keys to Alice and Bob. The second stage is Commit stage, where Alice sends Bob its commitment for a private message *m* ∈ *M* and secretly holds an opening value. The third stage is Open stage, where Alice sends Bob the original message *m* ∈ *M* along with the opening value, so that Bob can verify that the message committed in the first stage was indeed *m* ∈ *M*.

**Definition 9 (commitment function).**

First we define the commitment function *F* : ({0,1}^*n*^ × {0,1}^*n*^) → ({0,1}^*n*^ × {0,1}^*n*^), defined as *F*(*c*, *x*) = (*φ*, *δ*); here *φ* = *F*_*k*_(*m*, *x*) = *g*^*m*^*h*^*x*^  (mod *p*) and *δ* = *x* − *c* is the difference vector.

**Definition 10 (commitment protocol).**

Commitment protocol *π* is a scheme (for a message space *M*) defined by a triple (Setup, Commit, Open) such that

- (*p*, *q*, *g*, *h*) ← Setup(·) generates the public commitment key,
- for any *m* ∈ *M*,(*φ*, *δ*) ← Commit_(*p*, *q*, *g*, *h*)_(*m*) is the commitment/opening pair for *m*,
- Open_(*p*, *q*, *g*, *h*)_(*φ*, *δ*) → *m* ∈ *M* ∪ {*ε*}, where *ε* is returned if (*φ*, *δ*) is not a valid commitment to any message.

To set the system parameters, Trent executes the following procedure.

*Setup Procedure*

(1)* Trent* generates two prime numbers *p* and *q* such that *p* = 1  (mod *q*).

(2)* Trent* finds a random generator *g* ∈ *G*_*q*_∖{1}, where *g* ∈ *G*_*q*_ is a subgroup of the order *q* in *Z*_*p*_^*∗*^.

(3)* Trent* computes an element *h* = *g*^*a*^ ∈ *Z*_*p*_^*∗*^∖{1}, where *a* ∈ *Z*_*q*_ that is randomly chosen (*h* is a generator element of *G*_*q*_).

(4)* Trent* sends the system parameters (*p*, *q*, *g*, *h*) to Alice and Bob.

*Commit Procedure*. To commit to a message *m* ∈ *M*_*k*_⊆*Z*_*q*_ in the message space *M*_*k*_ ⊂ {0,1}^*k*^,* Alice* encodes the message into a codeword *c* = *g*(*m*) ∈ *C*⊆{0,1}^*n*^, chooses a random witness *x* ∈ *X*_*n*_⊆*Z*_*q*_ in the witness space *X*_*n*_ ⊂ {0,1}^*n*^, and then computes the commitment *F*(*c*, *x*) = (*g*^*c*^*h*^*x*^, *x* − *c*) = (*φ*, *δ*). The commitment is sent to* Bob*.

*Open Procedure*. To open the commitment (*φ*, *δ*),* Alice* reveals the witness *x*′, which is in proximity to the original *x* using some metric distance (e.g., Hamming distance *H*(*x*, *x*′) ≤ *t*_*σ*_). Using the difference vector *δ* the witness *x*′ restores the codeword *f*(*c*′) = *f*(*x*′ − *δ*) = *f*((*x*′ − *x*) + *c*) and then translates *x*′′ = *δ* + *f*(*c*′). Then* Bob* computes the commitment *φ*′ = *F*_*k*_(*f*(*c*′), *x*′′) and verifies $\varphi^{\prime }\overset{?}{=}\varphi$. In case of failure, the commitment will not open using *x*′. Otherwise, the commitment is successfully opened and therefore the secret message is *m* = *m*′ = *g*^−1^(*f*(*c*′)).

## 4. Security Properties and Analysis of the Proposed Scheme

Let *π* = (Setup, Commit, Open) be a commitment scheme, and its security properties are (i) correctness, i.e., for every message the commitment generated is valid, (ii) hiding, where any attacker cannot learn information from the commitment c about the message m with any advantage (perfect) or with a negligible advantage, and (iii) binding, where the message *m* is uniquely bound to *c* (perfect) or finding another message with the same commitment has negligible probability of success. In further analysis, we assume that both the codeword *c* and the witness *x* are drawn randomly from the finite set {0,1}^*n*^.

**Definition 11 (correctness).**

A commitment protocol *π* defined by the quadruplet (*p*, *q*, *g*, *h*) is correct if, for all messages *m* ∈ *M*, Open_(*p*, *q*, *g*, *h*)_(Commit_(*p*, *q*, *g*, *h*)_(*m*)) = *m*.

The hiding property of the biometrical scheme describes the resilience of the system against adversarial attempts performed by impostor* FakeBob* to crack codeword *c* or the witness *x*. We allege that impostor* FakeBob* knows *F* and can access the commitment (*φ*, *δ*).

The binding property represents the resistance of the system against adversarial attempts by an impostor *FakeBob*^*∗*^ to guess a codeword *c*′ with *H*(*x*, *x*′) ≤ *t*_*σ*_, such that *F*_*k*_(*c*, *x*) = *F*_*k*_(*c*′, *x*′) = *φ*, for some *x*, *x*′ ∈ *X*.

For hiding and binding, we have two different adversaries [51]:

- the* unhider* U, which plays the hiding game and has two abstract procedures, one to choose a pair of messages and another to guess which of the two messages corresponds to a given commitment;
- the* binder* B, which plays the binding game and has only a procedure to output two different pairs (message, opening value) that bind to the same commitment.

A commitment protocol satisfies the hiding security property if no adversary exists such that the probability of winning the hiding game is (significantly) better than a blind guess [51]. If this is true, the committer is guaranteed that no information can be inferred by the commitment itself.

**Definition 12 (hiding).**

Let *π* = (Setup, Commit, Open) be a commitment protocol. Then we can define the hiding properties for adversary U as Pr(*G*_*π*_^*H*^(*n*) = 1) = 1/2.

*Hiding Game*. The hiding game *G*_*π*_^*H*^ runs as follows:

(1) The adversary U is given the output of Setup procedure and asked to choose two messages.

(2) The game randomly selects one of them and calls Commit procedure to compute its commitment.

(3) The adversary U is asked to guess which one of the two messages the commitment corresponds to.

(4) The game outputs 1 if the guess of the adversary U is correct.

A commitment protocol satisfies the binding security property if no adversary exists such that the probability of winning the binding game is higher than negligible [51]. If this is true, the receiver is guaranteed that the value committed cannot be changed.

**Definition 13 (binding).**

Let *π* = (Setup, Commit, Open) be a commitment protocol. Then one can define the binding properties for each adversary B as Pr(*G*_*π*_^*B*^(*n*) = 1) = 0.

*Binding Game*. The binding game *G*_*π*_^*B*^ runs as follows:

(1) The adversary B is given the output of Setup procedure and asked to bind two messages to the same commitment value.

(2) The game outputs 1 if the two messages differ and the commitment is valid for both the messages, that is, if both can be verified by calling the Open procedure.

## 5. Application of the Method in EEG-Based Biometric System

**Figure 1:**
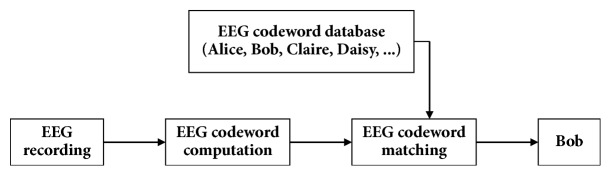
EEG-based user identification/authentication framework.

Here we present the biometric cryptosystem using the EEG signals. Its implementation consists of the system initialization stage, the enrolment stage, and the authentication stage as represented in Figure 1.

At the start of enrolment (see Algorithm 3), the user EEG biometrics is acquired, and feature extraction is performed using the EEG encoding algorithm, which outputs a 400-bit EEG code. We use the EEG features derived from the covariance matrix of EEG data from different EEG channels in the 10–20 international system. The covariance matrix is calculated from *N* channels in matrix as follows:

(3) $$\begin{array}{c} cov\left(\;X\;\right)=\frac{1}{N}\overset{N}{\underset{k=1}{\sum }}\left(\;X_{i,k}-X_{i}\;\right)\left(\;X_{j,k}-X_{j}\;\right), \end{array}$$

where *X*_*i*_ holds the mean of all observations in the respective EEG channels.

Next, we compute *z*-scores of the values in the covariance matrix as follows:

(4) $$\begin{array}{c} z_{i,j}=\frac{cov_{i,j}-\left(1/N\right)\sum_{i=1}^{N}cov_{i,j}}{\sqrt{{\sum_{i=1}^{N}\left(cov_{i,j}-\left(1/N\right)\sum_{i=1}^{N}cov_{i,j}\right)}^{2}/\left(N-1\right)}}; \end{array}$$

here cov_*i*,*j*_ is an element of the covariance matrix.

And perform normalization of *z*-score values of the covariance matrix into the range [0,1] as follows:

(5) $$\begin{array}{c} Z_{norm}=\frac{Z-\min\left(Z\right)}{\max\left(Z\right)-\min\left(Z\right)}. \end{array}$$

Finally, we perform the binarization of data using thresholding as follows:

(6) $$\begin{array}{c} Z_{bit}\left(\;i,j\;\right)=\left\{\begin{array}{cc} 0, & \left[z_{i,j}<0.5\right]\\ 1, & \left[z_{i,j}\geq 0.5\right]; \end{array}\right. \end{array}$$

here [·] is the Iverson bracket operator.

The result is a matrix that contains binary codeword of 400 bit length (obtained from 20 × 20 covariance matrix). The procedure is summarized in Algorithm 2.

At the same time, a random cryptographic key *κ* ∈ {0,1}^*k*^ is prepared using a BCH(…, *k*) error correction encoded function {0,1}^*k*^ → *C*. The result is a codeword *c* ∈ BCH(…, *k*), which is combined with reference EEG code (both have 400 bits of length).

Authentication phase is described in Algorithm 4. The input EEG biometric *B*_EEG_ is acquired from a person, resulting in a test EEG code *x*_test_. The test EEG code *x*_test_ with “exclusive OR” denoted as ⊕ extracts the codeword $\overset{⌢}{c}=\left(x_{test}⊕x_{ref}\right)⊕c$. Once it is extracted, the error correction decoded function of BCH(…, *k*) is used to compute $f\left(\overset{⌢}{c}\right)=f\left(x_{test}⊗\delta \right)$. Function$f\left(\overset{⌢}{c}\right)$ is used to compute $x_{test}^{\prime }=\delta ⊗f\left(\overset{⌢}{c}\right)=x_{ref}\left(c⊕f\left(\overset{⌢}{c}\right)\right)$. Nonvalid user will receive a codeword $f\left(\overset{⌢}{c}\right)$, such that $H\left(f\left(\overset{⌢}{c}\right),c\right)>t_{\sigma }$. Then $\varphi^{\prime }=F_{k}\left(f\left(\overset{⌢}{c}\right),x_{test}^{\prime }\right)$ is computed and matched against the stored *φ*. If *φ*′ = *φ*, then the sample *x*_test_ is accepted and the key *κ* is released. Otherwise, the identity of a person is rejected.

**Figure 2:**
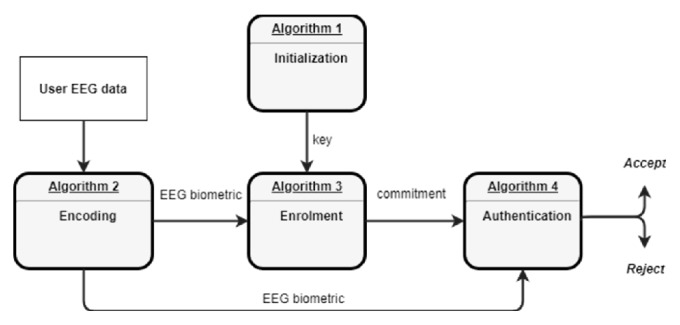
Summary of the proposed EEG biometric scheme.

The biometric scheme is summarized in Figure 2.

## 6. Experimental Results and Discussion

**Figure 3:**
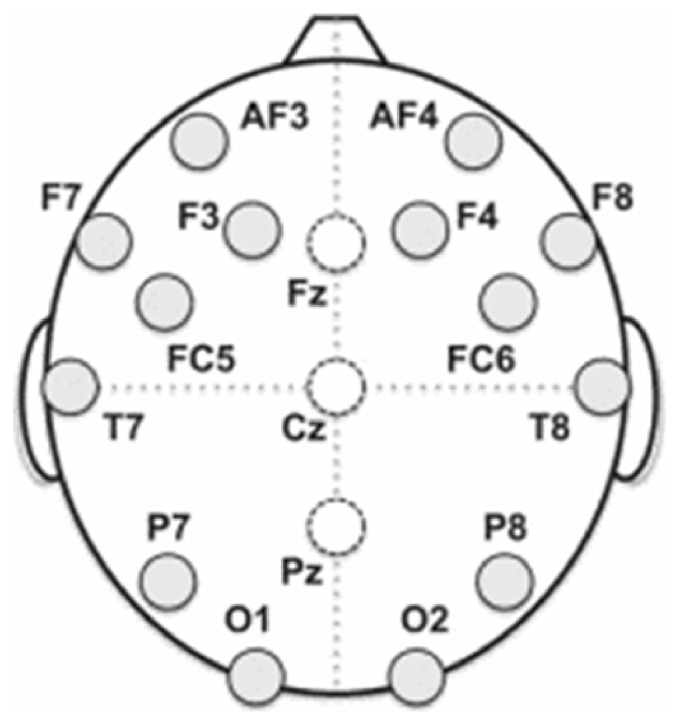
Electrode locations for collection of EEG data.

The implementation of the proposed scheme was made in MATLAB 8.6.0.267246 (R2015b) on an Intel (R) Core (TM) i5-4590 CPU (x64), running at 3.30 GHz with 12 GB of RAM in Windows 10 Enterprise ver. 1709. For the performance evaluation, we have used a dataset that consists of 65 EEG samples from 42 different subjects, where each sample consisted of 1000 signal values. The number of subjects satisfies the condition of Lazar et al. [52], who stated that studies using data collected from 20 or more participants are more convincing than those performed with a lesser number of participants. The EEG data we use in this study was collected from 42 healthy adults. During data collection, the subjects were instructed to lie still on a table and breathe normally. The data was collected using a medical-grade EEG device from the electrodes attached to subjects following the international 10–20 standard, which are depicted as circles in Figure 3. The sampling rate was 256 s^−1^.

To perform code matching, we computed the Hamming distance between two EEG codewords *A* and *B* as follows:

(7) $$\begin{array}{c} H=\frac{1}{n}\overset{n}{\underset{i=1}{\sum }}\left(\;code\left(\;A_{i}\;\right)⊕code\left(\;B_{i}\;\right)\;\right); \end{array}$$

here code(*A*_*i*_) and code(*B*_*i*_) are the *i*th bit in EEG codes of persons *A* and *B*, respectively.

**Figure 4:**
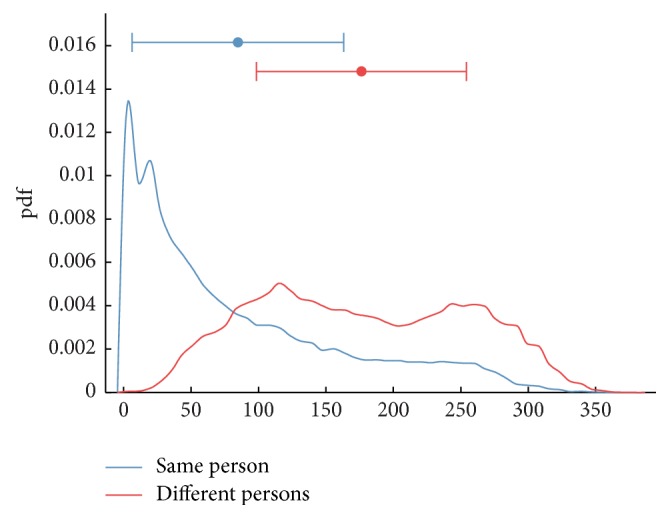
Probability density functions Hamming distances between the same person and the different persons.

The intraperson Hamming distances have been computed using EEG samples from the same subjects, while the interperson Hamming distances were computed using samples from different subjects. We carried out 65 comparisons for the same subjects and 118,335 comparisons between different subjects. The result of the probability distribution function (pdf) of the intraperson and interperson Hamming distances is shown in Figure 4. One can see that up to 87 bits of error (intersection of both graphs) are tolerated.

We use the following scenarios as suggested by Gui et al. [53].

**Scenario 1 .**

The aim is to identify correctly each of the 42 subjects participating in the study. The training and testing datasets include data from all 42 subjects and the classification outcome belongs to one of 42 classes.

**Scenario 2 .**

The aim is to identify one subject versus all other 41 subjects. There are only two classes: positive (target subject) and negative (all other subjects). The training dataset was combined using the data from all subjects and the performing resampling so that both classes are balanced.

*Evaluation*. Following the suggestion of Jorgensen and Yu [54], we use False Accept Rate (FAR), False Reject Rate (FRR), and Equal Error Rate (EER) as key effectiveness metrics of the biometric system. FAR and FRR describe whether the system correctly identifies the subject. ERR specifies the error rate where the values of FAR and FRR become equal. The metrics are calculated as follows:

(8) FRR=FRAA,FAR=FAIA;

here |FR| is the number of false rejections, that is, falsely rejecting a verification attempt of a valid subject, |AA| is the number of authorized attempts, |FA| is the number of false acceptances, i.e., falsely accepting the claim of an impostor as a valid user, and |IA| is the number of attempts by an impostor.

The performance is evaluated using the correct classification rate (CCR) as follows:

(9) $$\begin{array}{c} CCR=\frac{\left|C\right|}{\left|T\right|}; \end{array}$$

here |*C*| is the number of correct classification decisions and |*T*| is the number of trials.

EER is defined as a unique point where FRR is equal to FAR. A lower EER indicates a more accurate system.

(10) $$\begin{array}{c} EER=FAR\left(\;T^{*}\;\right)=FRR\left(\;T^{*}\;\right); \end{array}$$

here *T*^*∗*^ = argmin⁡(|FAR(*T*) − FRR(*T*)|)

This ensures that the threshold found will satisfy the equality condition between FRR and FAR as closely as possible.

**Figure 5:**
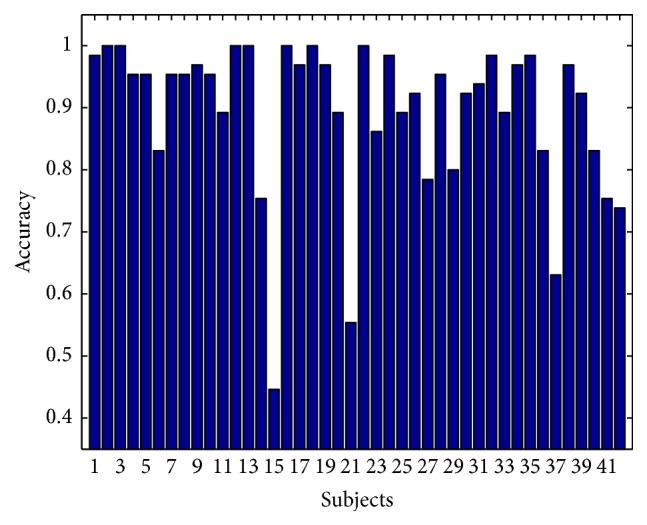
Subject-wise correct classification rate.

We have implemented both Scenarios 1 and 2 testing, as subjected by Gui et al. [53]. In Scenario 1, CCR for each of the subjects is presented in Figure 5.

**Figure 6:**
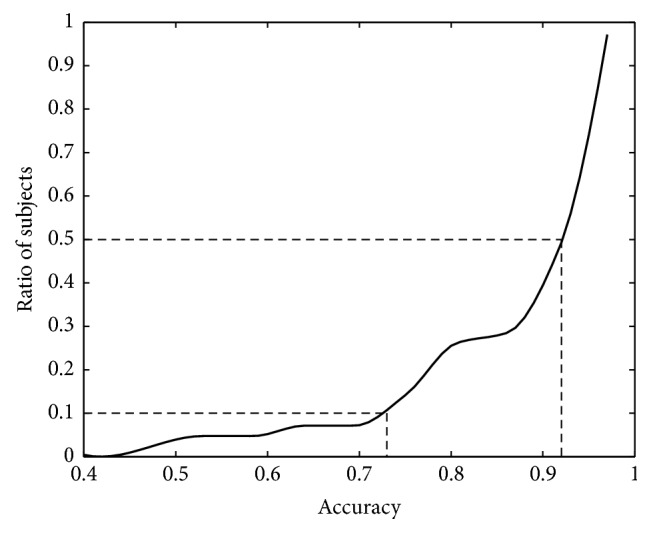
Cumulative distribution plot of accuracy distribution in subject classification.

Note that while the overall accuracy is quite good (mean accuracy 0.895), for some of the subjects, it was quite low (e.g., only 0.446 for subject 15). This result may have been caused by the infamous BCI illiteracy effect [55]. Nevertheless, when inspecting the cumulative distribution plot of accuracy distribution (see Figure 6), we can see that 50% of subjects have accuracy higher than 0.93, while only 10% of subjects have accuracy lower than 73%.

As accuracy data is not normally distributed, the Fisher *Z*-transformation was applied to calculate population mean and standard deviation, yielding the mean accuracy of 0.892 with standard deviation of 0.135.

**Figure 7:**
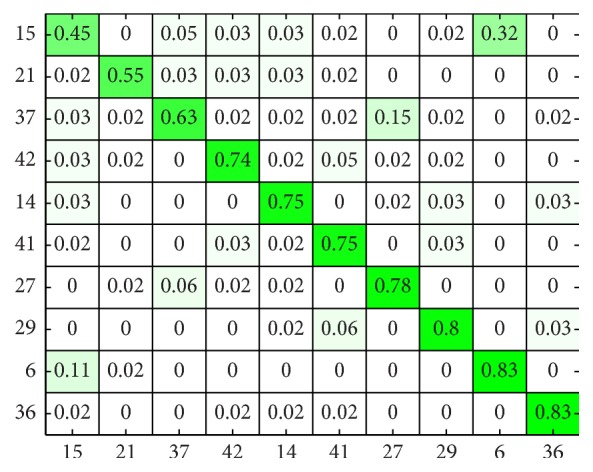
Subject-wise confusion matrix of classification results in Scenario 1: 10 worst performing subjects.

**Figure 8:**
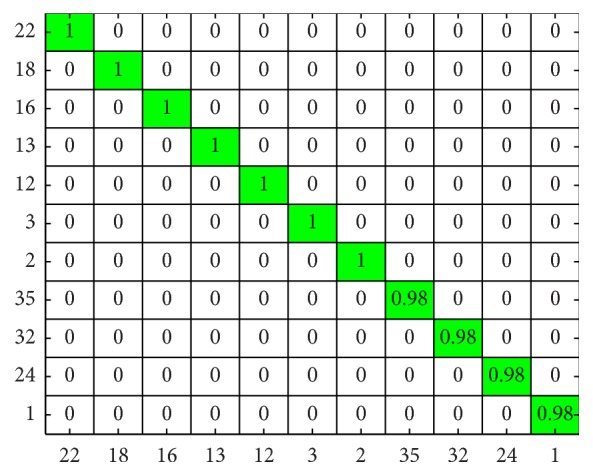
Subject-wise confusion matrix of classification results in Scenario 1: 10 best performing subjects.

The subject-wise confusion matrix is presented in Figures 7 and 8. As the number of subjects is too high for meaningful visualization, the confusion matrix was sorted according to its diagonal value (correct hits), and the values for only 10 worst performing subjects (Figure 7) and 10 best performing subjects (Figure 8) are shown.

**Figure 9:**
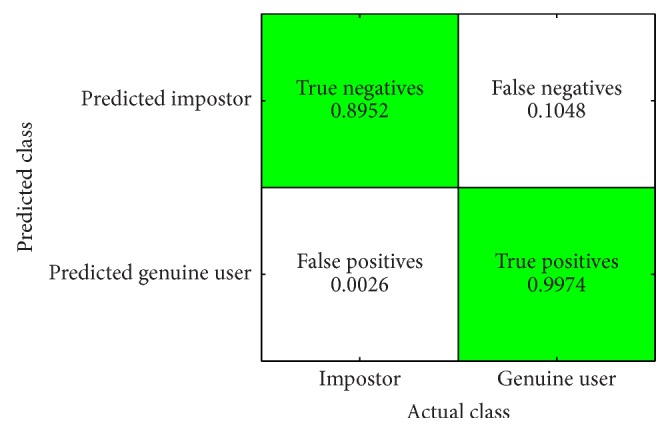
Confusion matrix of classification results in Scenario 2.

For Scenario 2, the confusion matrix is presented in Figure 9. We can see that True Positive Rate (TPR) is 0.9974. We have evaluated the confusion matrix statistically using the McNemar test. Critical value at 95% significance level is 3.8415. McNemar chi-square with Yates correction is 0.001, while *p* = 0.966. Therefore, the results are significant at alpha = 0.05 level.

**Figure 10:**
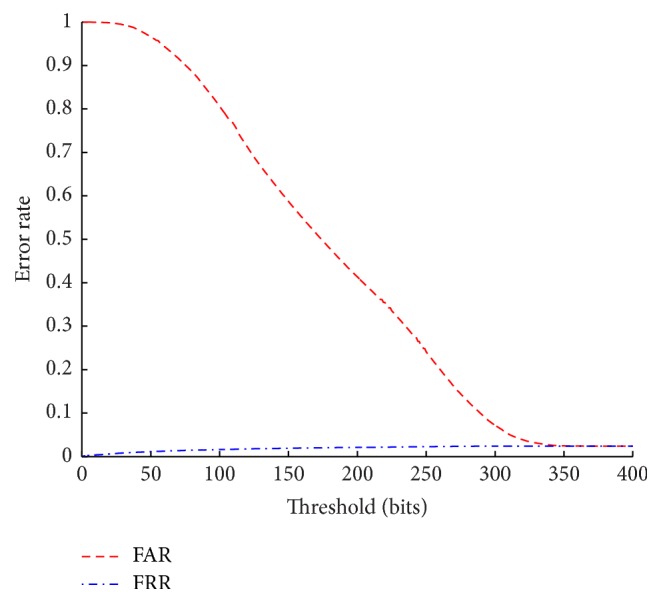
FAR and FRR of the proposed EEG biometric system.

The values for FAR, FRR, and ERR are represented in Figure 10.

The Area Under Curve (AUC) is calculated as the area under the Receiver Operating Characteristic (ROC) [56] curve and represents discrimination, that is, the ability of the classifier to discriminate between a positive example and a negative example.

We have achieved the following results, which are summarized in Table 1.

**Figure 11:**
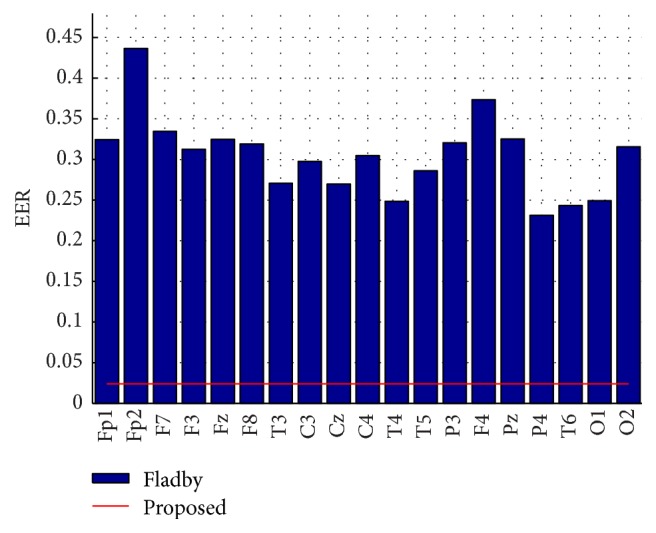
Comparison of EER of our method and Fladby's method [27] for each EEG channel.

*Comparison*. In Table 2 and Figure 11, we compare our results with those of Fladby [27]. Note that Fladby used a simple EEG reading device (Neurosky ThinkGear) with only one channel of EEG data (Fp1), which may be affected by eye artefacts. Sampling frequency was only 128 Hz, and 20 seconds of signal samples for each of eight different tasks was used for authentication, which is unpractical for many applications. Nevertheless, the method of Fladby [27], which employs widely used power spectral features of EEG bands, can be considered as a baseline, against which our method could be compared. We have thoroughly replicated the conditions of the experiment by Fladby on our dataset, using the same number of samples (2560) for each snippet of subject EGG data and a feature based distance metric to discriminate between genuine and fraudulent authentication results, and calculated the EER value. Note that our method uses all 20 EEG channels of the 10–20 international system, while Fladby used only one EEG channel. Nevertheless, we have replicated the calculations of the Fladby's method on each EEG channel to make a fair comparison. The results are presented in Table 2 as well as in Figure 11. Fladby's method achieved mean ERR of 0.3059, while the Fp1 channel originally used by Fladby achieved an ERR of 0.2945, and best ERR was achieved using the P4 channel (0.2283). Note that we could not apply our method on Fladby's data, because it is not available.

Based on the presented comparison, we can claim that the proposed method achieved better results for subject authentication than the Fladby [27] method.

## 7. Conclusion

This paper presents a secure cryptographic authentication scheme for EEG-based biometrics based on the fuzzy commitment scheme and the error-correcting Bose-Chaudhuri-Hocquenghem (BCH) codes. The EEG features are derived from the covariance matrix of EEG data from different EEG channels in the 10–20 international system. The biometric system was evaluated using the EEG dataset obtained from 42 subjects. The experimental results show that the system can generate up to 400 bits of cryptographic key from the EEG codes, while tolerating up to 87 bits of error. The performance of the biometric cryptosystem is an Equal Error Rate (EER) of 0.024, True Positive Rate (TPR) of 0.9974, and Area Under Curve (AUC) of 0.927.

## Figures and Tables

**Algorithm 1:**
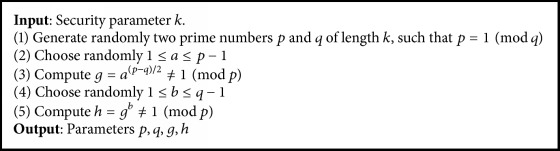
Initialization.

**Algorithm 2:**
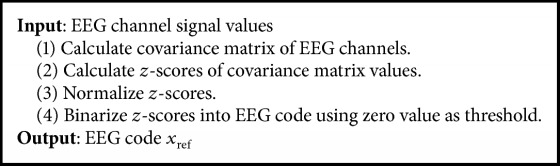
Encoding.

**Algorithm 3:**
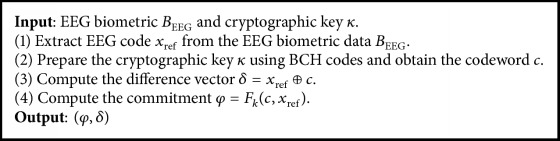
Enrolment.

**Algorithm 4:**
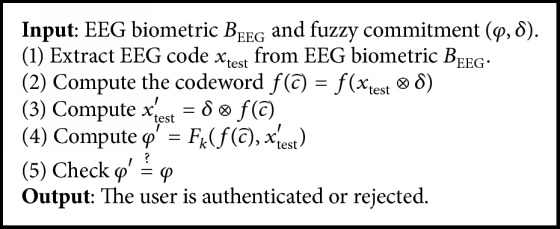
Authentication.

**Table 1:** Summary of classification results.

TAR	FRR	ERR	AUC	TPR
0.8952	0.026	0.024	0.9271	0.9974

**Table 2:** Comparison of the proposed method with the Fladby's method [27].

EER (proposed method + our dataset)	EER (Fladby method + Fladby dataset)	EER (Fladby method + our dataset)
0.024	0.2142	0.3059 (mean, all channels)
		0.2945 (Fp1)
		0.2283 (best, P4)
